# A Highly Sensitive and High-Resolution Resonant MEMS Electrostatic Field Microsensor Based on Electrostatic Stiffness Perturbation

**DOI:** 10.3390/mi14081489

**Published:** 2023-07-25

**Authors:** Xiangming Liu, Shanhong Xia, Chunrong Peng, Yahao Gao, Simin Peng, Zhouwei Zhang, Wei Zhang, Xuebin Xing, Yufei Liu

**Affiliations:** 1 State Key Laboratory of Transducer Technology, Aerospace Information Research Institute, Chinese Academy of Sciences, Beijing 100190, China; liuxiangming18@mails.ucas.edu.cn (X.L.); crpeng@mail.ie.ac.cn (C.P.); gaoyahao18@mails.ucas.ac.cn (Y.G.); pengsimin18@mails.ucas.ac.cn (S.P.); zhangzhouwei15@mails.ucas.ac.cn (Z.Z.); zhangwei178@mails.ucas.ac.cn (W.Z.); xxb2608@163.com (X.X.); liuyufei21@mails.ucas.ac.cn (Y.L.); 2 School of Electronic, Electrical and Communication Engineering, University of Chinese Academy of Sciences, Beijing 100049, China

**Keywords:** electrostatic field sensor, MEMS, resonator, frequency output

## Abstract

This paper proposes a highly sensitive and high-resolution resonant MEMS electrostatic field sensor based on electrostatic stiffness perturbation, which uses resonant frequency as an output signal to eliminate the feedthrough interference from the driving voltage. The sensor is composed of a resonator, driving electrode, detection electrode, transition electrode, and electrostatic field sensing plate. The working principle is that when there is an electrostatic field, an induction charge will appear at the surface of the electrostatic field sensing plate and induce electrostatic stiffness on the resonator, which will cause a resonant frequency shift. The resonant frequency is used as the output signal of the microsensor. The characteristics of the electrostatic field sensor are analyzed with a theoretical model and verified by finite element simulation. A device prototype is fabricated based on the Silicon on Insulator (SOI) process and tested under vacuum conditions. The results indicate that the sensitivity of the sensor is $0.1384\sqrt{\mathrm{Hz}}/\left(\mathrm{kV}/m\right)$ and the resolution is better than 10 V/m.

## 1. Introduction

Electrostatic field measurement technology is widely used in various fields, such as aerospace, meteorology, power grids, and the petroleum and petrochemical industries [1,2,3,4,5,6], and electrostatic field sensors play an important role in the measurement of electrostatic fields. With the development of Micro-Electro-Mechanical System (MEMS) technology, electrostatic field sensors based on MEMS have become one of the research hotspots in the field of electrostatic field detection due to their small size, low power consumption, and mass manufacturing. 

The MEMS electrostatic field sensors that have been reported can be classified into three categories according to their measurement principles, namely induction charge [7,8,9,10,11], steered electrons [12], and electrostatic force [13,14,15]. The induction charge MEMS electrostatic field sensors are the most mature, with the best electrostatic field resolution of 30 V/m [11]. The principle of the MEMS electrostatic field sensor based on induction charge is that the electrostatic field distribution around the sensing electrode is modulated by the shielding electrode, which vibrates forth and back periodically. Thus, a current is generated at the sensing electrode, and its amplitude changes with the intensity of the electrostatic field. However, there is a feedthrough between the sensing electrode and driving comb due to the AC driving signal, and a large zero-point output due to the DC driving signal. Therefore, the sensitivity and resolution of the electrostatic field sensor based on induction charge are difficult to improve.

However, there are still some detection applications that require better sensitivity and resolution in the electrostatic field measurement. To improve the sensitivity and resolution, Yang proposed a MEMS electrostatic field sensor with a shielding beam, aiming at reducing the feedthrough between the driving comb and sensing electrode and reducing the zero-point output; it had sensitivity of 0.2 mV/(kV/m), a resolution of 40 V/m, and a feedthrough signal of 9.8 mV [7]. Moreover, Liu proposed wafer-level vacuum packaging for an MEMS electrostatic field sensor, which had sensitivity of 0.16 mV/(kV/m), a resolution of 30 V/m, and a feedthrough signal of 4.2 mV under the condition of applying 5 V DC and 50 mV AC driving voltages [11]. The feedthrough signal was reduced due to the lower driving voltage. 

The resonant MEMS sensors with frequency as an output signal are characterized by a high resolution, high repeatability, high sensitivity, high accuracy, long-term stability, and quasi-digital outputs; they are widely used in various fields [16,17,18,19,20]. However, there are few reports on the resonant MEMS electrostatic field sensor based on frequency as the output signal. In this paper, a highly sensitive and high-resolution resonant MEMS electrostatic field sensor based on electrostatic stiffness perturbation is proposed. Different from the electrostatic field sensors based on induction charge, in which the resonator and shielding electrode are driven to modulate the electrostatic field distribution around the sensing electrode, the resonant MEMS electrostatic field sensors with frequency as the output signal convert the measured electrostatic field into stiffness, which affects the resonant frequency of the resonator, to achieve electrostatic field measurement. 

## 2. Structure Design and Work Principle

**Figure 1:**
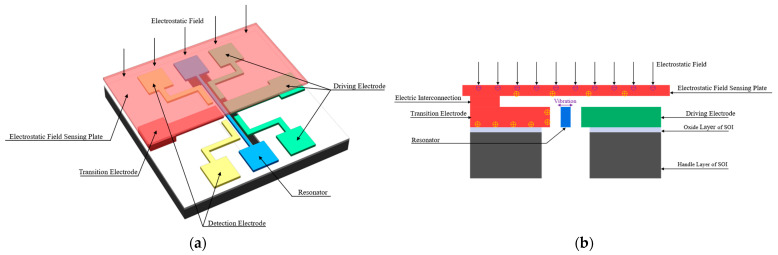
A schematic view of the proposed electrostatic field sensor. (**a**) A 3D schematic view of the proposed electrostatic field sensor; (**b**) a cross-section schematic view of the proposed electrostatic field sensor.

A schematic view of the proposed electrostatic field sensor is shown in Figure 1. The structure is composed of a resonator, a driving electrode, a detection electrode, a transition electrode, and an electrostatic field sensing plate. The electrostatic field sensing plate is an external metal plate or a cap on the metal packaging of the sensor that is electrically interconnected with the transition electrode. A parallel plate capacitor is formed between the transition electrode and the resonator to induce stiffness perturbation. When the sensor is placed in the electrostatic field, the positive and negative charges in the electrostatic field sensing plate and transition electrode are separated; however, the overall charge of the electrostatic field sensing plate and the transition electrode remains electrically neutral. The negative charge is concentrated at the upper surface of the electrostatic field sensing plate, and the positive charge is concentrated at the lower surface of the electrostatic field sensing plate, the lower surface of the transition electrode, and the sidewall of the transition electrode opposite to the resonator, as shown in Figure 1b. The positive charge induced by the electrostatic field at the sidewall of the transition electrode opposite to the resonator produces an electrostatic stiffness perturbation to the resonator, thus causing the resonant frequency of the resonator to shift. The measurement of the electrostatic field is achieved by detecting the resonant frequency shift of the resonator.

**Figure 2:**
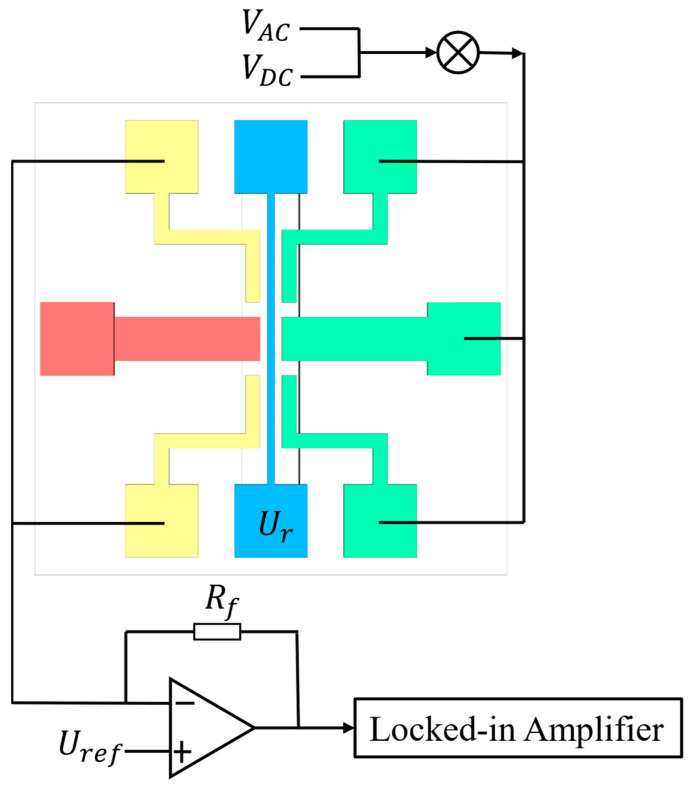
Schematic view of driving and detection.

In this paper, the resonator is driven by electrostatic driving, and the driving signal is a mixed signal of AC and DC. Capacitance detection is adopted to detect the vibration of the resonator. Due to the vibration of the resonator, the capacitance between the resonator and the detection electrode changes, and the induced current is generated at the detection electrode under the condition of a fixed voltage between the resonator and the detection electrode. A transimpedance amplifier is used to convert the induced current into a voltage signal, and the voltage signal is detected by a lock-in amplifier eventually. A schematic view of the driving and detection system is shown in Figure 2.

## 3. Theory

In this paper, a double-end fixed beam is adopted as a resonator. The resonant frequency is given by [21]

(1) $$f_{0}=\frac{22.4}{2\pi }\sqrt{\frac{Ew^{3}t}{12ml^{3}}}\;\;$$

where E is the Young’s modulus; m, w, t, l are the mass, width, thickness, and length of the resonator, respectively. The resonant frequency is changed due to the stiffness perturbation that is induced by the voltage between the resonator and transition electrode. The disturbed resonant frequency f and the resonant frequency variation Δf are given by

(2) $$f=\frac{1}{2\pi }\sqrt{\frac{k_{eff}+\Delta k}{m}}\;\;$$

(3) $$\Delta f=f-f_{0}=f_{0}\left(\sqrt{1+\frac{\Delta k}{k_{eff}}}-1\right)\approx \frac{1}{2}f_{0}\frac{\Delta k}{k_{eff}}\;$$

where $k_{eff}$ is the effective stiffness of the resonator, $k_{eff}=\frac{22.4^{2}Ew^{3}t}{12l^{3}}$; Δk is the stiffness perturbation.

According to Gauss Law, the amount of negative induction charge at the upper surface of the electrostatic field sensing plate is given by

(4) Q=εSE  

where ε is the dielectric constant, S is the effective area of the electrostatic field sensing plate, and E is the intensity of the electrostatic field component perpendicular to the electrostatic field sensing plate. Due to the parasitic capacitance $C_{pare}$, the positive induction charge generated by the electrostatic field sensing plate is only partially concentrated at the sidewall opposite to the resonator. Therefore, a charge transfer coefficient α is defined as the ratio of the charge at the sidewall of the transition electrode opposite to the resonator to the charge generated at the upper surface of the electrostatic field sensing plate, and the induction charge concentrating at the sidewall opposite to the resonator is defined as $Q^{\prime }$. Due to the fixed potential $U_{r}$ of the resonator in the application, the potential of the transition electrode can be calculated from the induction charge $Q^{\prime }$ and the capacitance between the transition electrode and the resonator $C_{p}$. A parallel plate capacitor model is adopted, and the voltage between the transition electrode and the resonator is given by

(5) $$V_{p}=\frac{Q^{\prime }}{C_{p}}=\frac{Q^{\prime }d}{\varepsilon A}\;\;$$

where A and d are the area and the gap between the transition electrode and the resonator, respectively. Therefore, the stiffness perturbation, Δk, generated by the measured electrostatic field is as follows:

(6) $$\Delta k=-\frac{\varepsilon AV_{p}^{2}}{d^{3}}=-\frac{\alpha^{2}\varepsilon SE^{2}}{Ad}\;$$

Therefore, the frequency output of the resonator relative to the measured electrostatic field is given by

(7) $$\Delta f=\frac{1}{2}f_{0}\frac{\Delta k}{k_{eff}}=-\frac{1}{2}f_{0}\frac{\alpha^{2}\varepsilon SE^{2}}{Adk_{eff}}\;$$

It can be seen that the sensitivity of the electrostatic field sensor is related to $f_{0}$, α, S, A, d, and $k_{eff}$ from Equation (7). To improve the sensitivity, we can increase α, S and decrease d. However, decreasing A may cause α to decrease, so the effect of A is studied in detail in Section 4.

**Figure 3:**
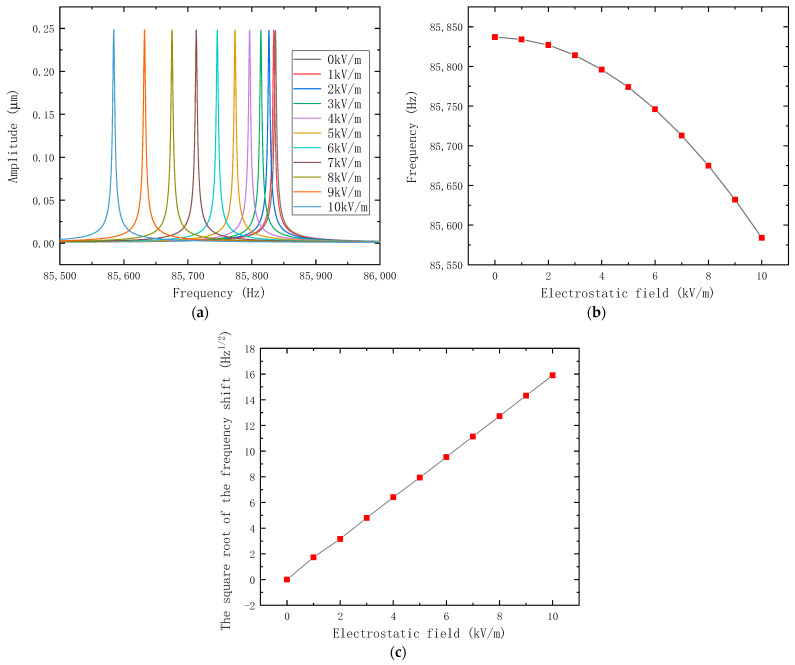
The theoretical calculation results of electrostatic field sensor. (**a**) The theoretical amplitude–frequency characteristic curves of the electrostatic field sensor under different electrostatic field intensities; (**b**) the theoretical frequency shift of the electrostatic field sensor; (**c**) the linearized theoretical output of the electrostatic field sensor.

Equation (8) represents a differential equation model of the proposed electrostatic field sensor whose structural parameters are shown in Table 1; it is used to simulate the output characteristics of the sensor, and the result is shown in Figure 3.

(8) $$m\ddot{x}+c\dot{x}+\left(k_{eff}+\Delta k\right)x=F\left(t\right)\;$$

where c is the damping, $c=\frac{m\omega_{0}}{Q_{qf}}$; $Q_{qf}$ is the quality factor, set to 30,000 based on previous experience; $\omega_{0}$ is the natural angular frequency; F(t) is the driving force. The charge transfer coefficient is set to 0.0001 according to the finite element simulation in Section 4.

## 4. Simulation

### 4.1. Optimization of Transition Electrode

**Figure 4:**
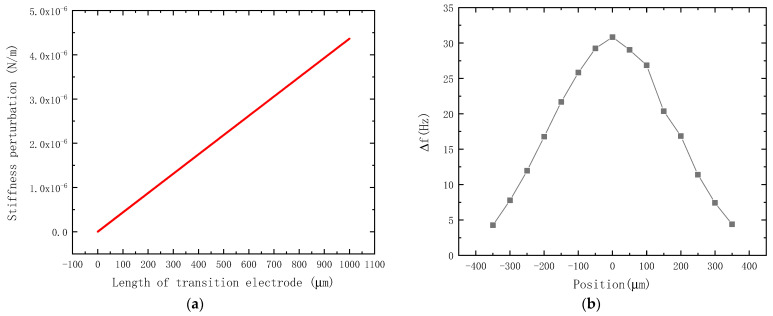
The optimization of the transition electrode by finite element simulation. (**a**) The stiffness perturbation versus the length of the transition electrode, where the length of the transition electrode represents the area of the transition electrode facing the resonator, due to the constant thickness of the SOI device layer; (**b**) the relationship between the frequency shift of the resonator and the position of the transition electrode, wherein the electrostatic field is 1000 V/m, the transition electrode is 180 μm, and the center of the transition electrode is at the middle of the resonator when the position equals zero.

The most important structural parameter of the proposed electrostatic field sensor is the charge transfer coefficient α according to Equations (6) and (7). Reducing the parasitic capacitance between the transition electrode and other structures can increase the charge transfer coefficient. In the design of an electrostatic field sensor, the distance between the transition electrode and other structures around it is increased to reduce the parasitic capacitance. In addition, the size and position of the transition electrode are optimized to increase the stiffness perturbation of the induction charge and the frequency shift of the resonator, as shown in Figure 4.

Figure 4a shows the relationship between the length of the transition electrode and the stiffness perturbation generated by the transition electrode to the resonator with the condition of the electrostatic field being 1000 V/m. According to Equation (6), the smaller the transition electrode is, the larger the stiffness perturbation is. However, the charge transfer coefficient α is related to the area of the transition electrode. The larger the transition electrode is, the larger the charge transfer coefficient is, due to the increase in the ratio of the capacitance between the transition electrode and the resonator $C_{p}$ to the overall capacitance $C_{p}+C_{para}$; therefore, an optimal value exists. It can be seen that the optimal transition electrode length is 1000 μm in the range of 0.1 μm to 1000 μm. However, considering the length of the resonator, driving electrode, and detection electrode, the length of the transition electrode is set to 180 μm.

Figure 4b shows the relationship between the output frequency shift of the resonator and the position of the transition electrode. It is shown that the optimal position is at the middle of the resonator, allowing us to obtain the maximum frequency shift.

### 4.2. The Output Characteristics of the Electrostatic Field Sensor

**Figure 5:**
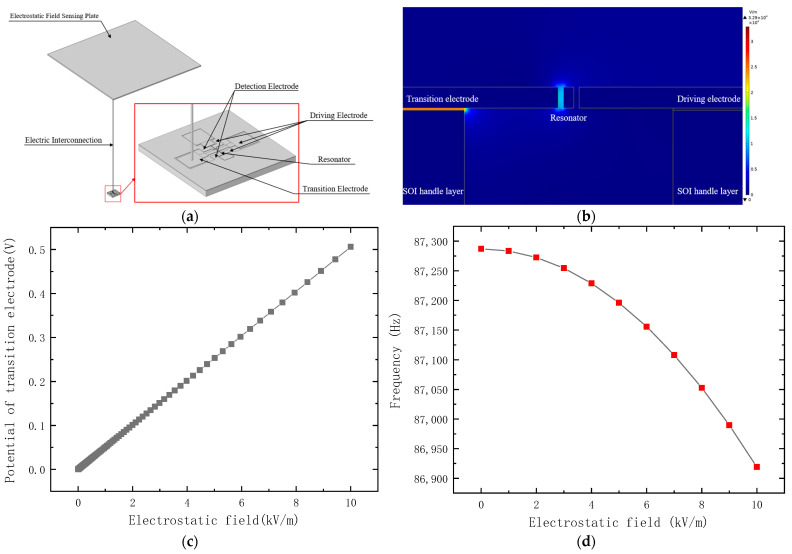

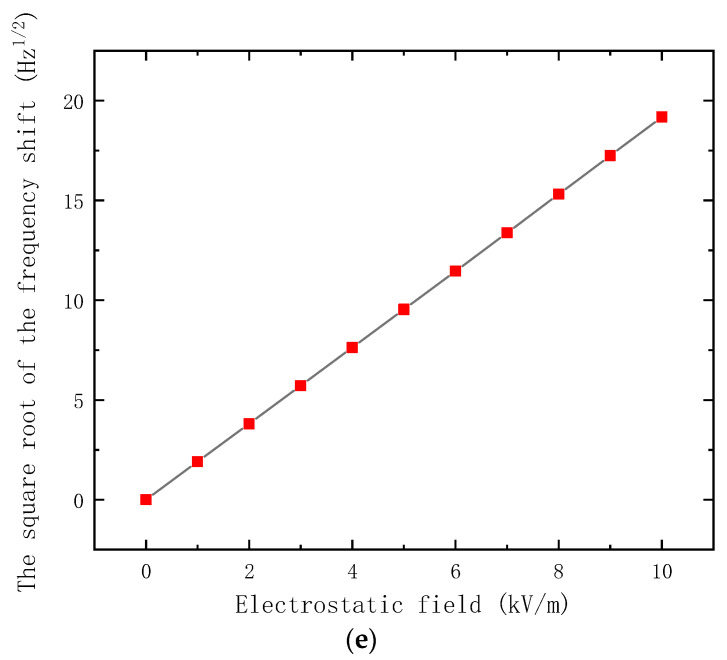
The simulation model and results of the electrostatic field sensor. (**a**) The finite element simulation model; (**b**) the electrostatic field distribution in the surroundings of the resonator under the condition of an electrostatic field of 1000 V/m; (**c**) the relationship between the potential of the transition electrode and electrostatic field, due to the induction charge concentration; (**d**) the simulated frequency shift of the electrostatic field sensor; (**e**) the simulated linearized output of the electrostatic field sensor.

A simplified finite element model is established according to the parameters shown in Table 1 using COMSOL, as shown in Figure 5a, wherein the electric interconnection is simplified as a copper metal cylinder, and the electrostatic field sensing plate is a $4\;{\mathrm{cm}}^{2}$ copper plate. The electrostatic field sensing plate, electric interconnection, and transition electrode are electrically floating, and the potential of the resonator and the rest of the structure is zero. Figure 5b presents the electrostatic field distribution in the surroundings of the resonator. It shows that the induction charge concentrates at the lower surface and sidewall opposite to the resonator of the transition electrode. Moreover, the induction charge at the lower surface of the transition electrode is greater than that at the sidewall opposite to the resonator, which is induced by the parasitic capacitance. According to Gauss Law, the induction charge Q and $Q^{\prime }$ are calculated. The result shows that Q is approximately 10,000 times higher than $Q^{\prime }$; therefore, the charge transfer coefficient is 0.0001. Figure 5c shows the relationship between the potential of the transition electrode and the measured electrostatic field. Figure 5d,e show the output characteristics, which are consistent with Equation (7) and Figure 3.

### 4.3. The Sensitivity Characteristics of the Electrostatic Field Sensor

Another strategy to improve the charge transfer coefficient is to change the potential of the resonator $U_{r}$, since the distribution of charge around the resonator is affected by the potential of the resonator. Due to the positive charge concentrating at the lower face of the transition electrode and sidewall opposite to the resonator, the lower the potential of the resonator is, the more the charge is concentrated at the sidewall of the transition electrode opposite to the resonator, which improves the charge transfer coefficient.

**Figure 6:**
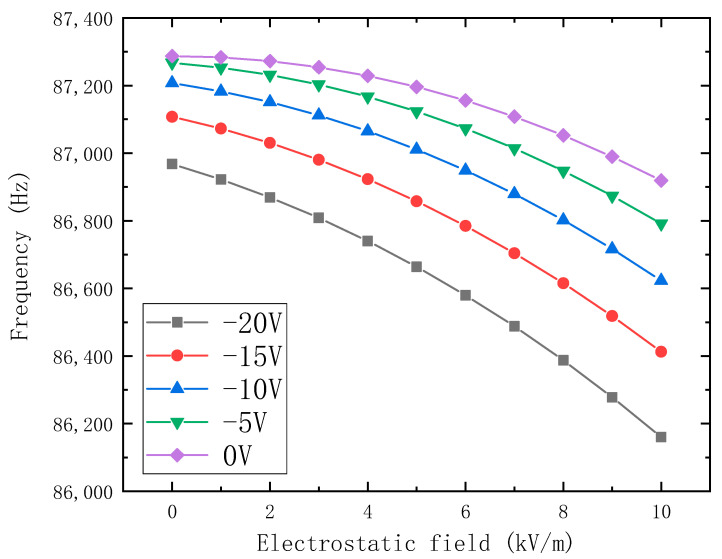
The relationship between the output frequency shift and the potential of the resonator.

Figure 6 shows that the output frequency is related to the different potential of the resonator in the condition range of 0 kV/m to 10 kV/m. The result shows that the lower the potential of the resonator is, the larger the output frequency shift is. Therefore, the sensitivity of the electrostatic field sensor can be improved by reducing the potential of the resonator.

## 5. Fabrication

**Figure 7:**
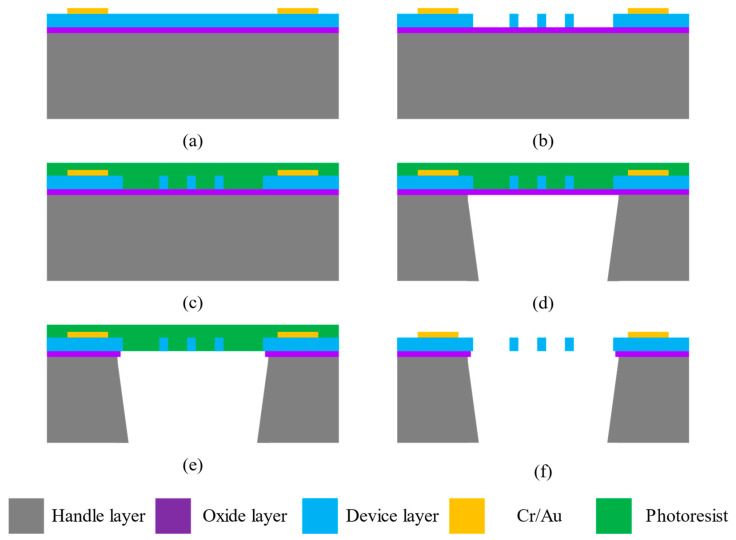
The main steps of the fabrication process.

The fabrication process of the electrostatic field sensing structure is based on SOI microprocessing technology in the laboratory. In the selected SOI, the thickness of the device layer is 20 μm, the resistivity of the device layer is 0.001–0.005 Ω·cm, the thickness of the oxide layer is 1 μm, the thickness of the substrate layer is 300 μm, and the resistivity of the substrate layer is 1–5 Ω·cm. The main steps, shown in Figure 7, are described as follows:

(a) Cr/Au pads are fabricated by the lift-off process on the surface of the device layer of the SOI;
(b) Deep reactive-ion etching (DRIE) is utilized to etch the device layer of the SOI to form the resonator, the driving electrode, the detection electrode, and the transition electrode;
(c) The photoresist is utilized to protect the microstructure by spin coating;
(d) DRIE is utilized to etch the handle layer of the SOI to form the vibration cavity;
(e) Reactive-ion etching (RIE) is utilized to etch the oxide layer of the SOI to release the movable structure;
(f) The protective photoresist is removed by fuming nitric acid and RIE with $O_{2}$.

**Figure 8:**
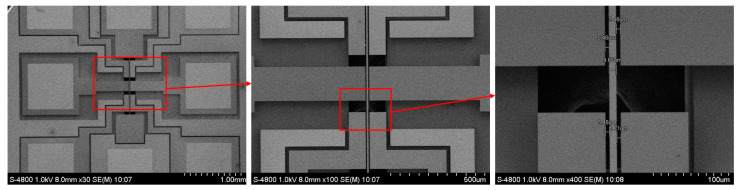
The SEM of the electrostatic field sensing structure.

Figure 8 shows the SEM of the electrostatic field sensing structure. The size of the chip is 5 mm×5 mm. The device layer of the SOI surrounding the transition electrode is etched to form a wider isolation groove; thereby, the parasitic capacitance is decreased and the charge transfer coefficient is increased.

## 6. Calibration System

**Figure 9:**
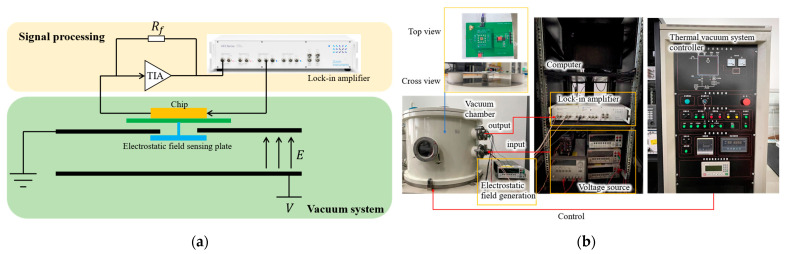
(**a**) A schematic of the test; (**b**) photographs of the testing system.

The calibration system of the electrostatic field sensor is shown in Figure 9, and it consists of two electrostatic field generation plates, a voltage source, a signal processing circuit, a lock-in amplifier, a computer, and a thermal vacuum system. The standard electrostatic field is generated by parallel plates. The gap between the two parallel plates is 2 cm, and it is supported by polytetrafluoroethylene columns, and a hole with a diameter of 3.5 cm in the center of the upper plate is created for the electrostatic field sensor. The voltage of the upper plate is zero, and the standard electrostatic field between the two plates is generated by adjusting the voltage between the two plates. The signal processing circuit mixes AC and DC driving signals for the chip and converts the output signals of the chip into voltage via a transimpedance amplifier. The signal output from the processing circuit is sent to the HF2LI lock-in amplifier for final extraction, and then it is displayed and recorded on the computer. Note that the electrostatic field sensor chip, the signal processing circuit, and the electrostatic field generation plates are placed in the vacuum chamber for testing.

## 7. Experiment

### 7.1. Amplitude–Frequency Characteristics

**Figure 10:**
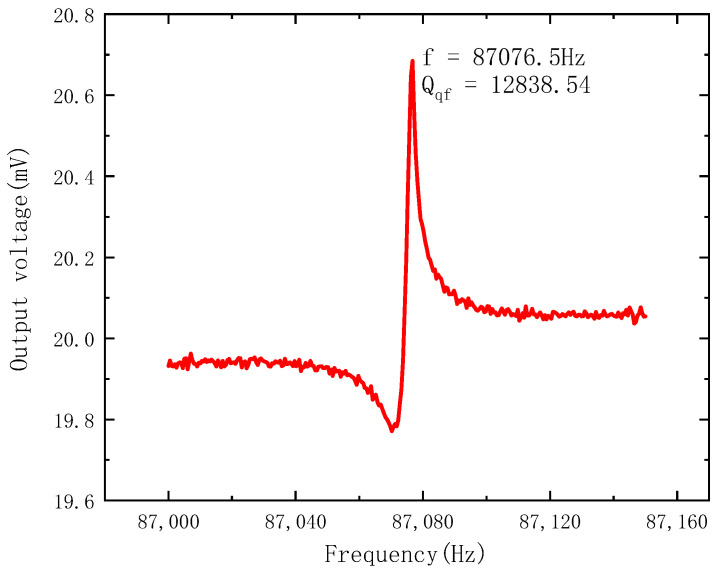
The amplitude–frequency characteristics of the electrostatic field sensor.

The tested amplitude–frequency response of the electrostatic field sensor is shown in Figure 10. The resonant frequency of the electrostatic field sensor is 87,076.5 Hz under the condition of 10 Pa pressure and without an electrostatic field. Compared with the simulated resonant frequency, the test result of the resonant frequency is in good accordance with the simulation result. The discrepancy is related to the fabrication process variations and the simulation deviation. The amplitude–frequency characteristic curve is asymmetrical due to the Fano resonance, which is caused by the phase difference between the vibration signal of the resonator and the feedthrough signal from the driving signal [22]. The quality factor is calculated as 12,838.54 according to the method in the literature [22].

### 7.2. Sensitivity Characteristics

**Figure 11:**
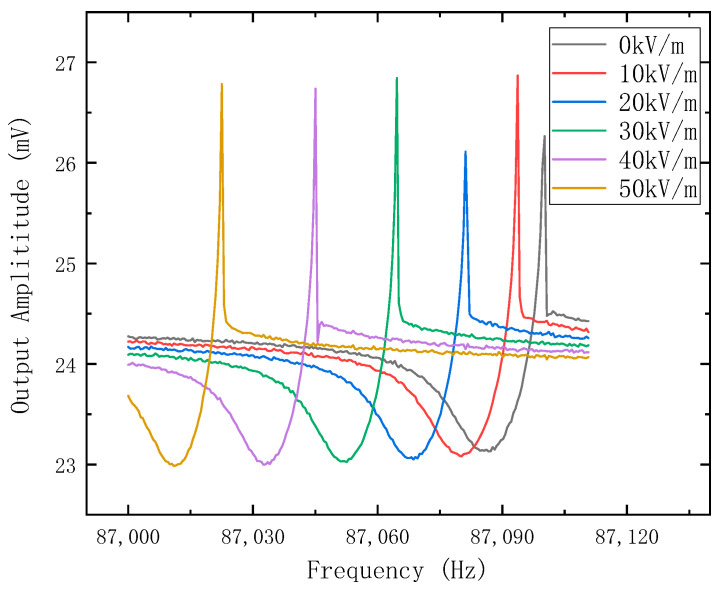
The amplitude–frequency responses versus the applied electrostatic field of the electrostatic field sensor.

**Figure 12:**
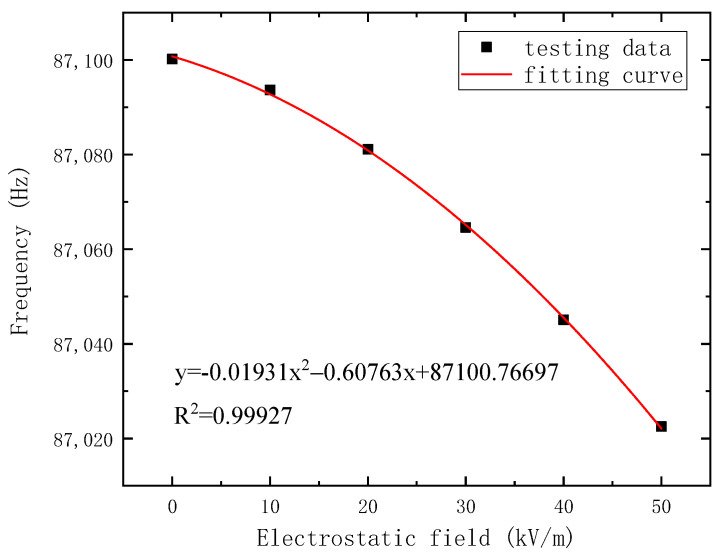
The result of the output resonant frequency versus the applied electrostatic field.

To improve the charge transfer coefficient and avoid the nonlinearity of the output signal, the voltage of the resonator is set to −10 V. Then, a 20 mV AC driving voltage is applied to the driving electrode. The frequency response versus the electrostatic field is shown in Figure 11. Next, the resonant frequency is extracted and processed, and the response result of the output frequency versus the applied electrostatic field is shown in Figure 12.

Comparing the theoretical and simulated output resonant frequency shifts, the frequency shift in the test result is small, due to the electrostatic field sensing plate connected with the transition electrode by the PCB; therefore, the parasitic capacitance is increased. According to Equation (7) and the test data, the charge transfer coefficient is approximately $1\times 10^{-6}$. Nonetheless, the average frequency shift of the proposed electrostatic field sensor reaches 1.57 Hz/(kV/m), and the frequency shift at 50 kV/m is 2.54 Hz/(kV/m).

In addition, the symmetry axis of the fitting curve is E=0 kV/m theoretically. However, the symmetry axis of the fitting curve is less than E=0 kV/m in the test. This is because both the driving and detection of the resonator introduce electrostatic stiffness, causing the initial frequency of the resonator, the frequency at E=0 kV/m, to shift. Therefore, there is a first-order term coefficient in the fitting curve.

**Figure 13:**
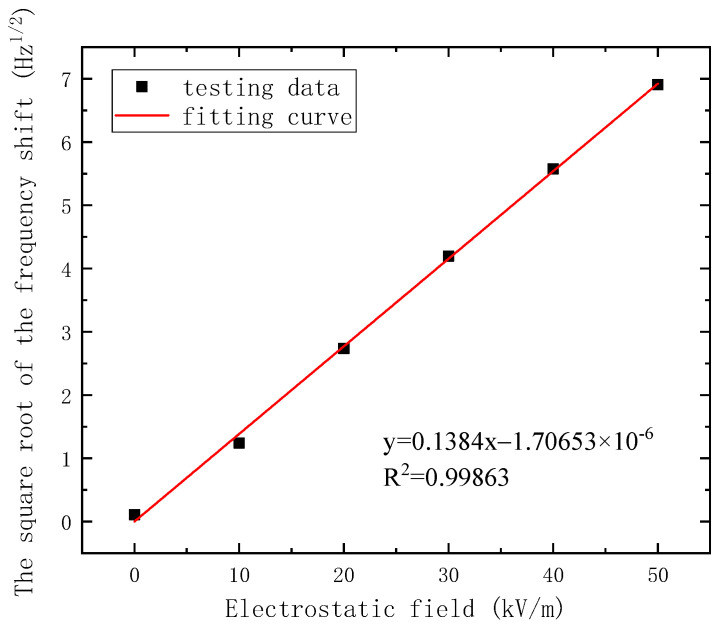
The linearization of the output resonant frequency.

Moreover, the linearization of the output resonant frequency is introduced, as shown in Figure 13, to quantitatively analyze the linearity of the sensor. The result shows that the sensitivity of the linearization is $0.1384\sqrt{Hz}/\left(kV/m\right)$, and the linearity is calculated as 2.49%. Compared with reported research [23], the sensitivity of the electrostatic field sensor considered in this paper has been improved by 10 times.

### 7.3. Resolution Characteristics

As the resonant frequency is the output signal, which is a type of quasi-digital output, the resolution of the proposed electrostatic field sensor is very high. The theoretical resolution is dependent on the minimum detectable frequency of the processing circuit. In this paper, the HF2LI lock-in amplifier used has high accuracy, and the minimum detectable frequency is 0.7 μHz; therefore, the theoretical detectable electrostatic field is $4.46\times 10^{-4}\;V/m$.

In the test, the standard electrostatic field is adjusted until an output frequency change is observed. The results show that the resolution of the electrostatic field sensor is better than 10 V/m.

## 8. Conclusions

In this paper, a highly sensitive and high-resolution resonant MEMS electrostatic field sensor based on electrostatic stiffness perturbation is proposed. Compared with traditional resonant MEMS electrostatic field sensors based on induction charge, the resonant frequency is used as the output signal in the proposed sensor. Based on the SOI process, a device prototype is fabricated and tested in a vacuum chamber. The experiment shows that the minimum detectable electrostatic field is better than 10 V/m, and the sensitivity is $0.1384\sqrt{Hz}/\left(kV/m\right)$.

## Figures and Tables

**Table 1:** Differential equation simulation parameters of the electrostatic field sensor.

Structural Parameters	Value
Length of the resonator	1000 μm
Width of the resonator	10 μm
Thickness of the resonator	20 μm
Mass of the resonator	$4.64\times 10^{-10}\;\mathrm{kg}$
Area of the electrostatic field sensing plate	4 ${\mathrm{cm}}^{2}$
Transition electrode length of facing resonator	180 μm
Gap	5 μm
Charge transfer coefficient	0.0001
Driving voltage	$10\;V_{DC}+20\;mV_{ac}$
Quality factor	30,000

## Data Availability

Not applicable.
